# Strategic surveillance plan for zoonotic leishmaniosis in Portugal

**DOI:** 10.1016/j.onehlt.2026.101572

**Published:** 2026-09-10

**Authors:** Clara M. Lima, Luís Cardoso, Joana Tavares, Mariana Portugal, Rita Gordo, Rui Ferreira, Nuno Santarém, Anabela Cordeiro da Silva

**Affiliations:** a Host-Parasite Interaction Group, Institute for Research and Innovation in Health, University of Porto. Porto, Portugal; b Microbiology Laboratory, Department of Biological Sciences, Faculty of Pharmacy, University of Porto, Porto, Portugal; c Department of Veterinary Sciences, and Animal and Veterinary Research Centre (CECAV), University of Trás-os-Montes e Alto Douro, Vila Real, Portugal; d Associate Laboratory for Animal and Veterinary Sciences (AL4AnimalS), Lisboa, Portugal; e School of Medicine and Biomedical Sciences (ICBAS), University of Porto, Porto, Portugal; f Centro de Recolha Oficial de Animais de Coimbra, Coimbra, Portugal; g Elanco Animal Health, Lisbon, Portugal; h Banco de Sangue Animal, Porto, Portugal

**Keywords:** Canine leishmaniosis, Feline leishmaniosis, *Leishmania infantum*, Multi-antigen ELISA, One Health, *Phlebotomus perniciosus*, Surveillance

## Abstract

**Background:**

*Leishmania infantum* is a vector-borne zoonotic parasite causing zoonotic leishmaniosis across Southern European countries, with poor prognosis for immunocompromised humans or animals. Improved monitoring and control of zoonotic leishmaniosis at the level of animal reservoir is critical to support One Health coordinated responses aligned with the objectives set out in the WHO roadmap for Neglected Tropical Diseases.

**Methods:**

We evaluate the suitability of a leishmaniosis surveillance system grounded on the network of public municipal animal shelters in Portugal (CROA), using dogs and cats as sentinels for sandfly exposure and *L. infantum* infection. Unowned dogs (*n* = 1194) and cats (*n* = 478) admitted at 34 CROA and four private shelters between 2021 and 2024, representing 90 municipalities distributed over six geographical regions of mainland Portugal, were clinically evaluated and serologically tested for antibodies to *Leishmania* parasites and *Phlebotomus perniciosus* salivary proteins by means of a multi-antigen enzyme linked immunosorbent assay (ELISA).

**Results:**

Canine (CanL) and feline (FeL) leishmaniosis seroprevalences were estimated at 11.8% and 5.0%, respectively. Serological evidence of exposure to *P. perniciosus* was found in 18.2% and 6.3% for dogs and cats, respectively. Significant regional differences in CanL and FeL seroprevalences were observed across the country, reflecting areas of differential endemicity. The study concluded that CROA network can substantially strengthen zoonotic leishmaniosis surveillance in Portugal, by enabling a geographically distributed sampling of animal reservoirs across multiple regions of mainland Portugal. When analysed using the proposed multi-antigen ELISA, these samples provide easily accessible, potentially useful and cost-effective epidemiological information.

## Highlights

• Leishmaniosis is an endemic vector-borne zoonotic disease in Portugal caused by *Leishmania infantum*.
• Portuguese CROA provide geographically distributed units for collecting epidemiological information on animal leishmaniosis and vector exposure.
• The SPLA- rK39 - SP03B multi-antigen ELISA enables high-throughput serological assessment of leishmaniosis and vector exposure in both dogs and cats.
• The observed geographical patterns provide epidemiological information that may support future surveillance and risk assessment strategies.
• Longitudinal sampling integrated with vector, environmental and human surveillance data are needed for operational surveillance capacity.

## Graphical abstract

**Figure:**
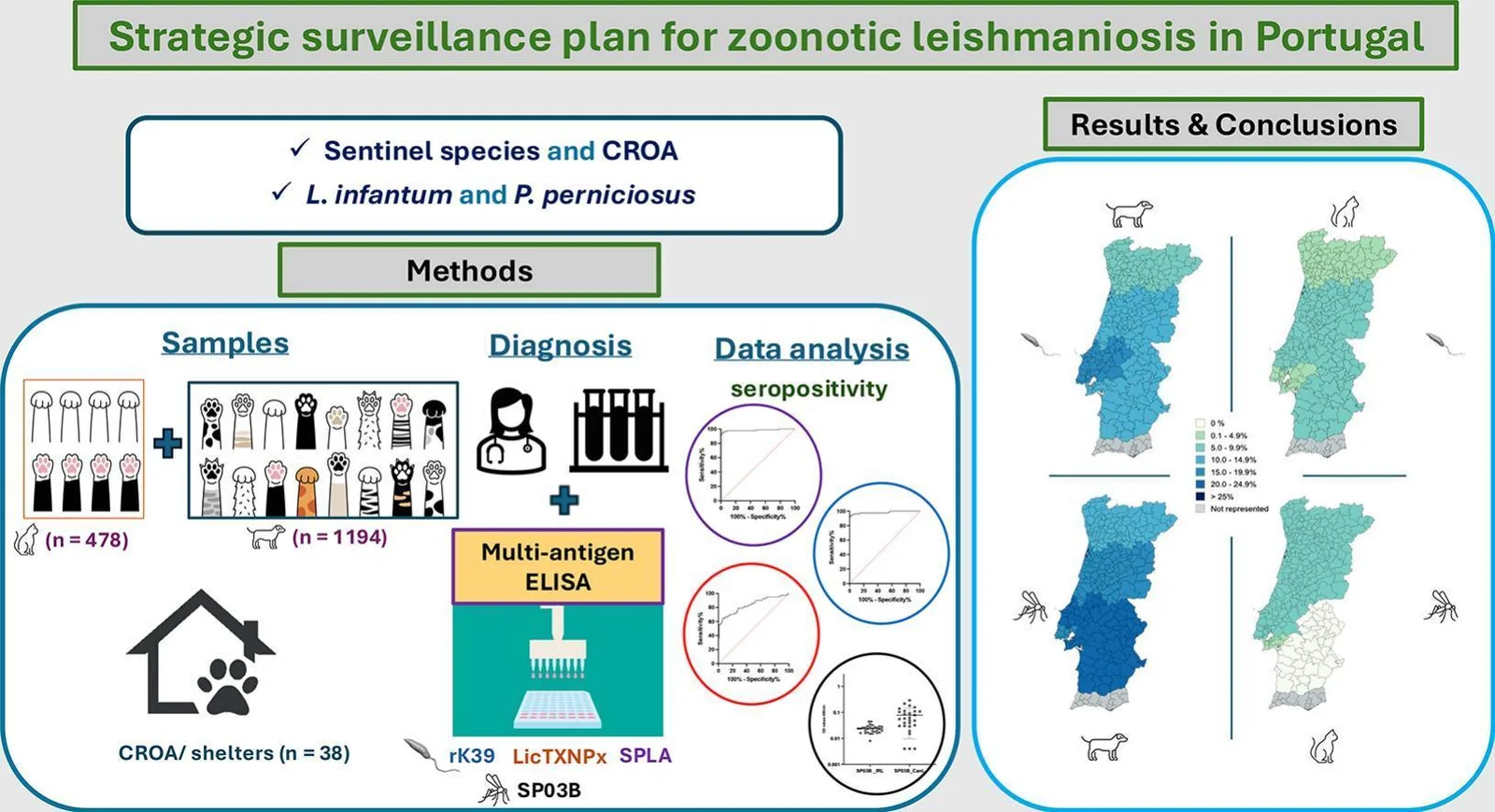

## 1 Introduction

In several southern European countries, leishmaniosis is a disease caused by the protozoan parasite *Leishmania infantum* (Kinetoplastida, Trypanosomatidae) that affects animals and humans. In this region, *L. infantum* transmission is zoonotic and primarily mediated by phlebotomine sandflies of the genus Phlebotomus. Infected animals can be infectious to vectors, and dogs are recognised as primary domestic reservoir hosts for human infection. Despite international commitments to end neglected tropical diseases (NTD), like the WHO roadmap for NTD [1], the burden of leishmaniosis remains underestimated due to irregular surveillance, fragmented reporting, and limited integration of animal, vector and human data. Leishmaniosis transmission is dynamic, and parasites are adapting to a gradually warmer climate and landscape changes, essential elements of sandfly dispersion and ecological adaptation [2], [3]. In Southern Europe, where L. *infantum* is endemic and causes zoonotic visceral leishmaniosis (VL), high levels of exposure among asymptomatic humans contrast with relatively low numbers of reported clinical cases [4], [5]. Nevertheless, for immunocompromised individuals and children are at risk [5]. In Portugal, human asymptomatic infection was estimated at 4.8% [4], and 221 VL cases were diagnosed during 2010–2020, predominantly among HIV-positive individuals and children [5]. Co-occurrence of immunosuppression and *Leishmania* infection often lead to severe disease, therapeutic failure, and poor outcomes [6], [7]. Since the prevalence of canine leishmaniosis (CanL), or canine infection with L. *infantum*, is a key determinant of human infection, monitoring CanL in endemic settings is a key strategy to prevent human infections or outbreaks [8], highlighting the need for a standardized and feasible strategy for monitoring animal reservoirs and support One Health surveillance. In fact, seroprevalence of CanL in Southern European countries remains high in many of its countries, with 23.3% of exposure estimated in Italy, 12.5% in Portugal, 12.3% Greece, and 7.7% in Spain (reviewed by Plesko et al. [9]). Also, and importantly, feline infection with *Leishmania* is being increasingly reported in Southern European countries. Most recent studies point to 17% seroprevalence [10]. Despite the alarming figures, felines´ contribution to sustained transmission, particularly in the absence of dogs, remains unclear.

Regardless both veterinary and public health concern surrounding CanL, the disease is not listed in the European Animal Health Law among those subject to harmonized prevention and control measures (EU Regulation 2016/429). While the most affected countries (i.e. Italy, Greece, Portugal, Albania) undergo surveillance and notification of animal and human cases under the directions of national and/or regional regulations [11], the absence of standardized procedures for surveillance and notification undermine regular reporting of human and animal leishmaniosis to WHO or the World Organization for Animal Health (WOAH), respectively [11].

In Portugal, *Leishmania* transmission occurs seasonally, potentially from May to November, mostly aligned with *Phlebotomus perniciosus* activity [12]. An increasing seroprevalence of CanL has been reported (e.g. 6.3% in 2009 to 12.5% in 2021, following national CanL prevalence studies among dogs attending veterinary centres) [13]. In a contrasting, yet equally relevant epidemiological scenario, 1 million unowned dogs and cats are estimated to roam freely [14], likely subject to higher exposure to vectors and *Leishmania* infection [15]. Each year, around 40,000 of these animals are admitted to Portuguese public animal shelters, known as “Centro de Recolha Oficial de Animais” (CROA) [16], forming a large and geographically diverse group that remains largely untapped for surveillance. Although national legislation recognises CanL as a zoonotic risk and supports surveillance, it primarily does so at operational level linked to rabies vaccination campaigns [17]. Upon a suspect clinical presentation of CanL, the municipal veterinarian (MV) informs the dog owner on the need to follow-up with diagnostic requirements, and initiates notification actions to the Veterinary Authorities. An annual report including such notifications is made public [17]. In practice, this framework underestimates true burden of infection because CanL prevalence is not reported; subclinical cases are not identified; diagnostic approaches are heterogeneous; and surveillance is biased towards the small fraction of the canine population that presents for rabies vaccination campaigns.

Effective surveillance of zoonotic leishmaniosis should allow comparison of epidemiological data over-time, help prevent outbreaks, avoid re-emergence of human disease in endemic regions and detect the introduction and establishment of imported and exotic *Leishmania*. Integrating robust and standardized serological data with epidemiological and clinical information improves awareness and preparedness, supports early outbreak detection and guides evidence based targeted control measures [18]. Since the Portuguese monitoring and surveillance of zoonotic leishmaniosis among domestic reservoirs is limited to passive case detection, the resulting data provides insufficient information to assess the burden of infection and its geographic distribution. Furthermore, this strategy does not support reliable assessment of epidemic risks or a potential zoonotic spill over.

In this study, our primary objective was to evaluate the feasibility of implementing a surveillance strategy for canine and feline exposure to *Leishmania* and its main phlebotomine vector in Portugal. To achieve this, the national CROA network was considered as a framework of operational epidemiological units, and a multi-antigen ELISA was utilized as a highly sensitive and specific diagnostic tool, suitable for high-throughput screening. We hypothesize that dogs and cats admitted at the geographically dispersed CROA can serve as complementary sentinel hosts, providing geographically distributed information on *Leishmania infantum* exposure and vector contact that could be incorporated into a broader One Health strategy for leishmaniosis surveillance and control.

## 2 Materials and methods

### 2.1 Ethics statement

This study received approval from the Animal Welfare and Ethical Commission for use of animals in research (ORBEA) from the School of Medicine and Biomedical Sciences (ICBAS), University of Porto, Portugal under the protocol n. 385/2020. Written informed consent was signed by the Veterinarian responsible for each animal facility.

### 2.2 Study concept and design

Municipal veterinarians (MV) are responsible for overseeing CROA in Portugal. At the onset of this study, 166 municipalities used licenced CROA to accommodate stray animals, representing 53.8% of the total number of Portuguese municipalities (*n* = 308) [19]. Following national regulation, CROA are responsible for collecting stray animals from public areas as a specific action to tackle zoonotic diseases and promote animal welfare [20], [21]. The MV were invited to take part in the study either by email or phone. If participation was declined or the municipality did not have a CROA, a local private animal shelter partnered with the municipality was contacted instead. Upon approval, a visit was arranged to collect blood samples and perform clinical examinations on resident dogs and cats, including stray cats admitted through trap–neuter–return (TNR) programs.

### 2.3 Canine and feline populations and study area

In total, 1194 dogs and 478 cats were sampled between 2021 and 2024 from 34 CROA and four private shelters located across mainland Portugal. These sampling sites represented 90 of the approximately 166 municipalities of mainland Portugal with access to a CROA. Geographical analysis of CanL and FeL seroprevalence was conducted according to the stratified Nomenclature of Territorial Units for Statistics (NUTS), a hierarchical system for dividing up European Union countries for administrative and statistical purposes [22]. To estimate seroprevalence at the level of standardized NUTS2 regions, and within the boundaries of each sampling site (NUTS level 3 or individual/adjacent municipalities), data was aggregated for NUTS2 regions and further disaggregated to NUTS3 or municipalities (whenever NUTS3 were not defined, e.g. in Lisbon NUTS2 and Setúbal Peninsula NUTS2).

Animals older than six months were eligible for inclusion. Dogs and cats that could not be safely restrained were excluded unless sedation or anaesthesia was required for other procedures, such as microchipping, neutering, or clinical treatment, which allowed blood sampling. Free-roaming animals with microchips registered outside the CROA municipality were excluded from that municipality or regional cohort.

### 2.4 Sample size

A sample size of 409 dogs would be required to determine CanL prevalence at a national level if 0.99 test sensitivity (Sn), 0.98 test specificity (Sp), 50% expected prevalence (to provide the highest sample size when the true prevalence is unknown), ± 5% absolute error and 0.95 confidence level (CI) are considered [23]. However, at each CROA, sampling could only be conveniently performed depending on the number of animals admitted at the visit's time point. Testing was conducted on all dogs when the shelter population totalled 30 or fewer. When the population exceeded this threshold, a random sample was selected for inclusion in the study. The sample size depended on the capacity to perform clinical evaluations and collect blood samples from these animals within the period allotted by the CROA responsible to perform the study. Feline sampling was limited by the number of cats available for adoption at each institution and/or the number of cats captured for TNR at the time of sampling.

### 2.5 Laboratory investigation

#### 2.5.1 Serological detection of antibodies to Leishmania in dogs and cats

Total immunoglobulin G (IgG) against L. *infantum* was measured in canine and feline sera using an in-house indirect multi-antigen enzyme-linked immunosorbent assay (ELISA) based on different *Leishmania*-specific antigens, including soluble promastigote *Leishmania* crude proteins (SPLA), recombinant *Leishmania* kinesin 39 (rK39) and L. *infantum* cytosolic tryparedoxin peroxidase (CPX), as previously described [24], [25]. Canine samples were tested for SPLA and rK39 seroreactivity according to the protocol described by Santarém et al. [24]. Seropositivity was considered for samples testing positive to SPLA and/or rK39. Feline samples were tested according to the protocol described by Lima CM et al. [26] and seropositivity was considered for samples testing triple positive to SPLA, rK39 and CPX.

For the same sample, total IgG to *Phlebotomus perniciosus* saliva were simultaneously detected using the recombinant salivary protein 03B (SP03B), kindly provided by Fabiano Oliveira from National Institute of Allergy and Infectious Diseases (NIAID), USA. The coating conditions were followed as previously described [27].

Each sample was simultaneously tested for all antigens in three technical replicates, under the same experimental conditions. Seroreactivity was determined for each sample and antigen by the average optical densities (OD) of at least two comparable assays. Each test included a species-specific positive control (serum sample from clinical cases of CanL and FeL with confirmed parasitological diagnosis of *Leishmania* infection) and blank samples. A non-related antigen composed of soluble proteins recovered from the ubiquitous gram-negative bacteria *Escherichia coli* (SECA) was introduced as internal control of reaction, and to address *Leishmania*- or *P. perniciosus*- specific seroreactivity. SECA production followed the protocol by Lima C et al. [28].

#### 2.5.2 Seropositivity cut-offs

For dogs, positivity thresholds for *Leishmania*-specific antigens were determined by receiver-operating characteristic (ROC) curve using two control groups: Group A (CanL-positive), including 81 clinically confirmed cases; Group B (CanL-negative) including 125 non-infected dogs from different geographical regions. Both clinical, parasitological and serological characterization of positive and negative CanL controls are described in Supplementary Fig. 1 and Supplementary Table 1. The derived sensitivity (Sn) and specificity (Sp) for SPLA, rK39 and SPLA and/or rK39 combination was determined from the ROC curve analysis and presented in Supplementary Table 2. Following comparison of the modified Yuden's index, positivity to SPLA and/or rK39 was selected for CanL serodiagnosis.

For cats, positivity thresholds for *Leishmania*-specific antigens were adopted from Lima CM et al. [26].

### 2.6 Statistical analysis

Statistical analyses were performed using IBM SPSS Statistics for Windows version 27.0 (IBM, Armonk, NY, USA), Epitools Epidemiological Calculators, Ausvet (available at: http://epitools.ausvet.com.au.) and MedCalc for Windows, version 19.4 (MedCalc Software, Ostend, Belgium; available at: https://www.medcalc.org/en/). Descriptive statistics was used to describe continuous variables in Group A and Group B (detailed in Supplementary Table S1). Comparisons between both groups was performed by Mann-Whitney *U* test. Statistically significant differences were assumed for *p* < 0.05. To evaluate leishmaniosis seropositivity from both geographical and clinical perspectives, proportions between groups of categorical variables – including exploratory variables such as location, signalment and clinical evaluation parameters – were compared using contingency tables. The Chi-squared test (χ^2^) or Fisher's exact test (FET) was applied as appropriate, considering 95% CI. Statistical significance was defined as *p* < 0.05.

Graphical representations were performed using GraphPad Prism software version 9 (San Diego, CA, USA). Maps were drawn in www.mapchart.net/portugal.html (accessed on 28 December 2025).

### 2.7 Seroprevalence analysis

Both CanL and FeL seroprevalence and exposure to *P. perniciosus* bites was calculated for dogs and cats as the number of seropositive animals divided by the total number of animals tested for each studied NUTS2, NUTS3 or municipality.

### 2.8 Clinical characterization of CanL and FeL cases

A physical examination was performed on all sampled animals. Seropositivity was associated to a body condition score, the presence of dermatological lesions, gastro-intestinal signs, neuro-muscular alterations, ophthalmological lesions, and systemic signs of disease.

## 3 Results

### 3.1 Geographical landscape of canine and feline leishmaniosis seroprevalence and associations between seropositivity and host factors

**Fig. 1:**
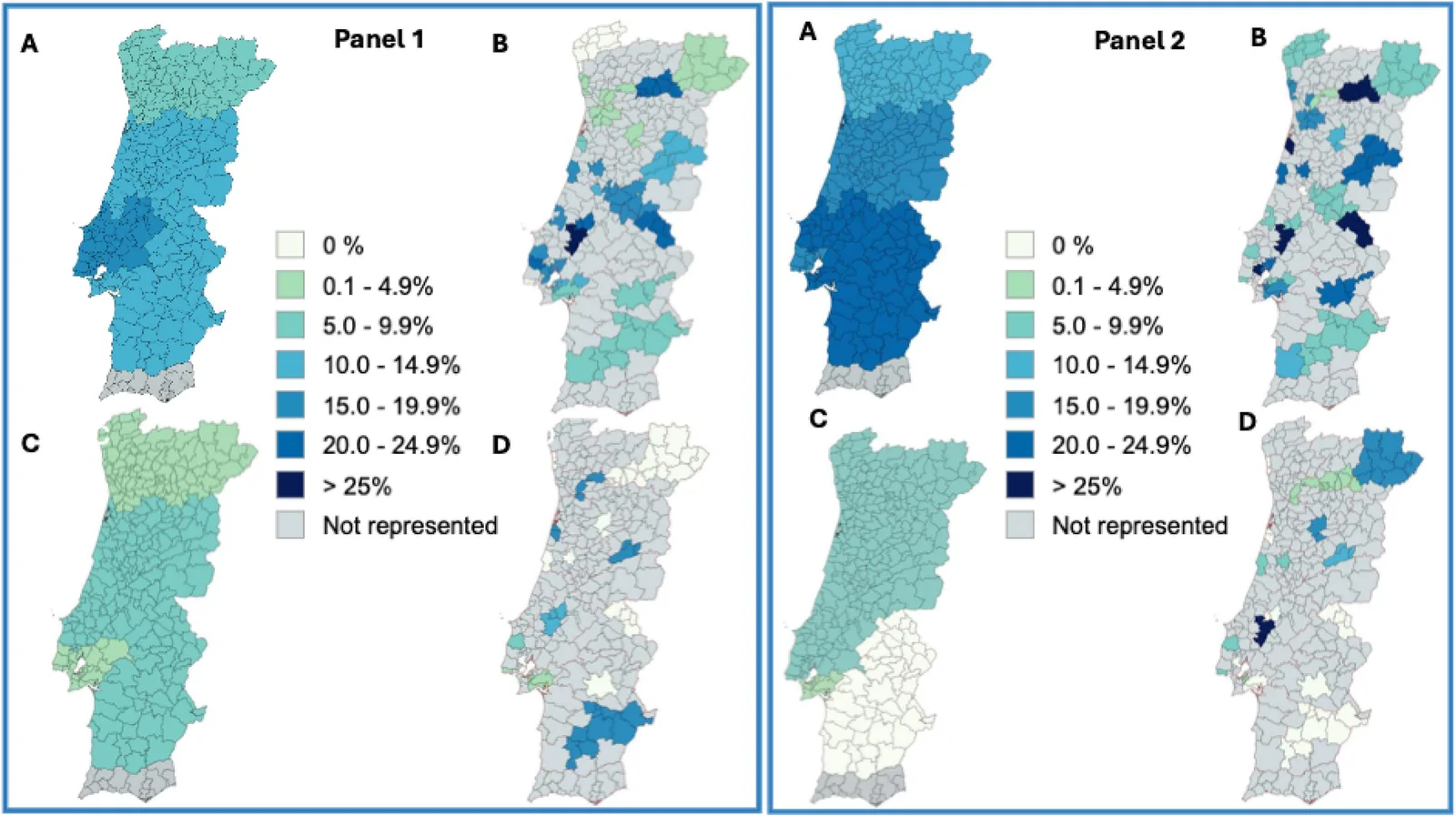
Geographical distribution of canine (CanL) and feline (FeL) leishmaniosis seroprevalence (%) and exposure to *Phlebotomus perniciosus* salivary protein (SP) 03B (%) in the studied population of dogs and cats. Seroprevalence data was produced using an enzyme-linked immunosorbent assay and is depicted by geographical regions of mainland Portugal, classified according to the Nomenclature of Territory Units for Statistics (NUT) classification 2, and its respective surveyed municipalities. Maps drawn in www.mapchart.net/portugal.html, accessed on 28 December 2025. Panel 1. Distribution of CanL and FeL seroprevalence (%) across mainland Portugal. (A) CanL seroprevalence across the NUTS2 regions of mainland Portugal. (B) CanL seroprevalence across the surveyed municipalities of mainland Portugal. (C) FeL seroprevalence across the NUTS2 regions of mainland Portugal. (D) FeL seroprevalence across the surveyed municipalities of mainland Portugal. CanL seropositivity was determined by an enzyme linked immunosorbent assay (ELISA) considering positivity to soluble promastigote *Leishmania* antigens (SPLA) and/or *Leishmania infantum* recombinant kinesin (rK39). FeL seropositivity was determined by an enzyme linked immunosorbent assay (ELISA) considering triple positivity to soluble promastigote *Leishmania* antigens (SPLA), *Leishmania infantum* recombinant kinesin (rK39) and L. *infantum* cytosolic tryparedoxin peroxidase (CPX). Panel 2. Distribution of canine and feline exposure to *P. perniciosus* bites (%) across mainland Portugal, measured by seropositivity to SP03B. (A) Canine seropositivity to SP03B across the NUTS2 regions of mainland Portugal. (B) Canine seropositivity to SP03B across the surveyed municipalities of mainland Portugal. (C) Feline seropositivity to SP03B across the NUTS2 regions of mainland Portugal. (D) Canine seropositivity to SP03B across the surveyed municipalities of mainland Portugal.

Table 1 and Fig. 1 summarise canine and feline seropositivity to L. *infantum* and exposure to *P. perniciosus* bites. Seroprevalence data is stratified by NUTS2 and NUTS3 regions, depicting the geographical distribution of leishmaniosis and vector-exposure across these two reservoir species in mainland Portugal.

**Table 1:** Seroprevalence of canine (CanL) and feline (FeL) leishmaniosis and exposure to *Phlebotomus perniciosus* bites across six regions of mainland Portugal, classified according to the Nomenclature of Territory Units for Statistics (NUTS) – levels 2 and 3 – and across the littoral and the interior regions of mainland Portugal. Leishmaniosis seroprevalence among canine (*n* = 1194) and feline (*n* = 478) hosts and canine (*n* = 995) and feline (*n* = 335) seropositivity to *P. perniciosus* salivary antigen SP03B were assessed in grouped dogs and cats according to their respective origin. Chi-square tests were used to evaluate associations between geographical variables and the proportion of seropositive results in the studied populations, assuming 95% confidence interval (CI) and statistically significant differences for *p* < 0.05. Comparison of canine (CanL) and feline (FeL) leishmaniosis and SP03B seropositivity between sympatric dogs and cats, sampled at the same time, was performed by Fischer's Exact Test assuming 95% confidence interval (CI) and statistically significant differences for *p* < 0.05.

NUTS Regions		No. of CanL^a^ seropositive dogs per region (%)^c^	No. of FeL^b^ seropositive cats per region (%)^c^	No. of sick CanL seropositive dogs (%)^c^	No. of sick FeL seropositive cats (%)^c^	No. of SP03B seropositive dogs (%)^c^	No. of SP03B seropositive cats (%)^c^
NUTS2	NUTS 3 or municipality						
**Norte** (PT11)							
Alto Minho (PT111)^1^		0/46 (0)	NT	0/46 (0)	NT	4/46 (8.7)	NT
Douro (PT11D)^2^		6/29 (20.7) d	0/37 (0) d	3/29 (10.3)	0/37 (0)	6/12 (50.0) d	1/29 (3.5) d
		*p* = 0.01^⁎d^				*p* = 0.001*^d^	
Terras de Trás-os-Montes (PT11E)^2^		1/42 (2.4) d	0/29 (0) d	0/42 (0.0)	0/29 (0)	3/42 (7.1) d	5/27 (18.5) d
		*p* = 1.0 d				*p* = 0.265 d	
Tâmega e Sousa (PT11C)^2^		2/45 (4.4) d	4/26 (15.4) d	2/45 (4.4)	3/26 (11.5)	2/45 (4.4) d	1/22 (4.5) d
		*p =* 0.2 d				*p* = 1.0 d	
Área Metropolitana do Porto (PT11A)^1^		4/86 (4.7)	0/6 (0)	1/86 (1.2)	0/6 (0)	15/85 (17.7)	0/5 (0)
**Total sympatric animals**		9/116 (7.8)	4/92 (4.3)			11/99 (11.1)	7/78 (9.0)
		*p =* 0.4				*p* = 0.805	
**Total Norte**		13/248 (5.2)	4/98 (4.1)	6/248 (2.4)	3/98 (3.1)	30/230 (13.0)	7/83 (8.4)
**Centro** (PT19)							
Região de Aveiro (PT191)^1^		2/33 (6.1) d	4/22 (18.2) d	0/33 (0)	4/22 (18.2)	15/33 (45.5) d	0/21 (0) d
		*p* = 0.387 d				*p =* 0.003*^d^	
Região de Coimbra (PT192)^1^		23/120 (19.2) d	0/33 (0) d	15/120 (12.5)	0/33 (0)	12/54 (22.2) d	1/12 (8.3) d
		*p* = 0.009*^d^				*p* = 0.683 d	
Viseu-Dão-Lafões (PT194)^2^		1/24 (4.2) d	0/23 (0) d	0/24 (0)	0/23 (0)	3/24 (12.5) d	2/12 (16.7) d
		*p* = 1.0 d				*p* = 1.0 d	
Beira Baixa (PT195)^2^		6/40 (15.0)	NT	1/40 (2.5)	NT	2/30 (6.7)	NT
Beiras e Serra da Estrela (PT196)^2^		9/88 (10.2) d	3/19 (15.8) d	5/88 (5.7)	2/19 (10.5)	19/88 (21.6) d	2/16 (12.5) d
		*p* = 0.694 d				*p* = 0.735 d	
Região de Leiria (PT193)^2^		1/9 (11.1)	NT	0/9 (0)	NT	0/9 (0)	NT
**Total sympatric animals**		35/265 (13.2) d	7/97 (7.2) d			49/199 (24.6) d	5/72 (6.9) d
		*p* = 0.193 d				*p <* 0.001* ^d^	
**Total Centro**		42/314 (13.4)	7/97 (7.2)	21/314 (6.7)	6/97 (6.2)	51/238 (21.4)	5/61 (8.2)
**Oeste e Vale do Tejo** (PT1D)							
Oeste (PT1D1)^2^		5/81 (6.2) d	2/39 (5.1) d	2/81 (2.5)	1/39 (2.6)	6/70 (8.6) d	2/30 (6.7) d
		*p* = 1.0				*p* = 1.0	
Lezíria do Tejo (PT1D3)^1^		12/38 (31.6) d	1/8 (12.5) d	5/38 (13.2)	1/8 (12.5)	14/30 (46.7) d	3/8 (37.5) d
		*p* = 0.668				*p* = 1.0	
Médio Tejo (PT1D2)^1^		12/53 (22.6) d	2/16 (12.5) d	3/53 (5.7)	0/16 (0)	3/31 (9.7) d	0/15 (0) d
		*p* = 0.724				*p* = 0.543	
**Total sympatric animals**		29/172 (16.9)	5/63 (7.9)			23/131 (17.6) d	5/53 (9.4) d
		*p* = 0.125				*p* = 0.263 d	
**Total Oeste e Vale do Tejo**		29/172 (16.9)	5/63 (7.9)	10/172 (5.8)	2/63 (3.2)	23/131 (17.6) d	5/53 (9.4) d
**Lisboa** (PT1B)							
Loures^1,^^e^		5/28 (17.9) d	0/26 (0) d	3/28 (10.7)	0/26 (0)	12/28 (42.9) d	0/21 (0) d
		*p* = 0.06				*p =* 0.005*	
VFX^1,^^e^		11/61 (18.0)	NT	6/61 (9.8)	NT	13/60 (21.7)	NT
Cascais^1,^^e^		0/17 (0.0) d	3/63 (4.8) d	0/17 (0.0)	2/63 (3.2)	1/17 (5.9) d	3/34 (5.9) d
		*p* = 1.0				*p* = 1.0	
Mafra^1,^^e^		6/25 (24.0)	NT	0/25 (0.0)	NT	NT	NT
**Total sympatric animals**		5/45 (11.1)	3/89 (3.4)			26/105 (24.8) d	3/55 (5.5) d
		*p* = 0.130				*p* = 0.013*^d^	
						
**Lisbon total**		22/131 (16.8)	3/89 (3.4)	9/131 (6.9)	2/89 (2.2)	26/105 (24.8) d	3/55 (5.5) d
**Península de Setúbal** (PT1B0)							
Alcochete and Montijo^1,c^		7/59 (11.9)	NT	3/59 (5.1)	NT	4/53 (7.5)	NT
Barreiro and Moita^1,c^		11/95 (11.6) d	1/63 (1.6) d	2/95 (2.1)	0/63 (0)	15/76 (19.7) d	1/22 (4.5) d
		*p* = 0.032*^d^				*p* = 0.04*^d^	
Palmela and Setúbal^1,c^		5/59 (8.5) d	1/31 (3.2) d	4/59 (6.8)	1/31 (3.2)	9/46 (19.6) d	0/28 (0) d
		*p* = 0.660				*p* = 0.025*^d^	
**Total sympatric animals**		16/154 (10.4)	2/94 (2.1)			24/122 (19.7) d	1/50 (2.0) d
		*p* = 0.021*				*p* = 0.001*^d^	
**Total Península Setúbal**		23/213 (10.8)	2/94 (2.1)	9/213 (4.2)	1/94 (1.1)	28/175 (16.0)	1/50 (2.0)
**Alentejo** (PT1C)							
Alentejo Litoral (PT1C1)^1^		1/14 (7.1)	NT	0/14 (0.0)	NT	2/14 (14.3)	NT
Baixo Alentejo (PT1C2)^2^		3/36 (8.3) d	3/19 (15.8) d	3/36 (8.3)	3/19 (15.8)	2/36 (5.6) d	0/18 (0) d
		*p* = 1.0				*p* = 1.0	
Alto Alentejo (PT1C3)^2^		3/13 (23.1) d	0/6 (0) d	1/13 (7.7)	0/6 (0)	23/116 (19.8) d	0/33 (0) d
		*p* = 0.532				*p* = 0.001* ^d^	
Alentejo Central (PT1C4)^2^		5/53 (9.4) d	0/12 (0) d	2/53 (3.8)	0/12 (0)	13/53 (24.5) d	0/10 (0) d
		*p* = 0.579				*p* = 0.195	
**Total sympatric animals**		11/102 (10.8) d	3/37 (8.1) d			21/102 (20.6) d	0/33 (0) d
		*p* = 1.0 d				*p* = 0.001* ^d^	
						
**Total Alentejo**		12/116 (10.3)	3/37 (8.1)	6/116 (5.2)	3/37 (8.1)	23/116 (19.8)	0/33 (0)
Littoral^1^		81/733 (11.1)	11/277 (4.0)	42/724 (5.8)	8/277 (2.9)	108/582 (18.6)	7/173 (4.0)
Interior^2^		60/461 (13.0)	13/201 (6.5)	19/470 (4.0)	9/201 (4.5)	73/413 (17.7)	14/162 (8.6)
		χ2 = 0.978, df = 1, *p* = 0.323 CI: −1.8 to 5.9%	χ2 = 1.515, df = 1 *p* = 0.218 CI: −1.5% to 7.1%	χ2 = 1.909, df = 1 *p* = 0.167 CI: −0.8% to 4.2%	χ2 = 0.864, df = 1 *p* = 0.353 CI: −1.8% to 5.7%	χ2 = 0.131, df = 1, *p* = 0.717 CI: −4.0% to 5.7%	χ2 = 3.024, df = 1, *p* = 0.110 CI: −0.7% to 10.3%
**Total seropositive** (%)		141/1194 (11.8)	24/478 (5.0)	61/1194 (5.1)	17/478 (3.6)	181/995 (18.2)	21/335 (6.3)
		χ2 = 17.755, df = 1, *p* < 0.001* CI: 3.9 to 9.3%		χ2 = 1.724, df = 1, *p* = 0.189 CI: −0.9 to 3.4%		χ2 = 27.509, df = 1, *p* < 0.001* CI: 8.03% to 15.20%	

VFX, Vila Franca de Xira; NT, not tested.

a Canine leishmaniosis, CanL, determined by seropositivity to soluble promastigote *Leishmania infantum* antigens (SPLA) and/or *Leishmania* recombinant kinesin 39 (rK39).

b Feline leishmaniosis, FeL, determined by the combined seropositivity to SPLA, rK39, and L. *infantum* recombinant cytosolic peroxiredoxin protein (CPX).

c (%), percentage of seropositivity.

e Identification of the Municipality covered by a local CROA whenever NUTS3 subdivision is not available.

d Sympatric animals, sampled at the same time point.

1 Littoral regions

2 Interior regions

A total of 1194 dogs were sampled, of which 733 (61.4%) originated from the littoral and 461 (38.6%) from the interior regions of mainland Portugal. Sampling encopasse the Norte (248/1194, 20.8%), Centro (314/1194, 26.3%), Oeste e Vale do Tejo (172/1194, 14.4%), Lisboa (131/1194, 11.0%), Península de Setúbal (213/1194, 17.8%) and Alentejo (116/1194, 9.7%) NUTS2 regions (Table 1).

Overall, CanL seropositivity determined by positivity to SPLA and/or rK39 was 11.8% (141/1194), with individual antigen seropositivity ranging between 10.2% (122/1194) for SPLA and 8.3% (99/1194) for rK39. Marked heterogeneity in CanL seroprevalence was observed across NUTS2 regions (Fig. 1 panel 1A) and within NUTS3 subregions or municipalities (Fig. 1 panel 1B). The highest values were detected in areas surrounding the Tagus River Valley, including the Oeste e Vale do Tejo and Lisboa NUTS2 regions, with an overall seroprevalence of approximately 17% (Table 1; Fig. 1 panel 1A). Within the Oeste e Vale do Tejo region, the NUTS3 subregions of Lezíria do Tejo and neighbouring Médio Tejo were identified as hotspots. Here, CanL seroprevalence reached 31.6% and 22.6%, respectively (Table 1; Fig. 1 panel 1B). The highest CanL seroprevalence within Lisboa NUTS2 region was identified at the municipality of Mafra, with 24.0% (Table 1; Fig. 1 panel 1B). The two neighbouring municipalities of Loures and Vila Franca de Xira present 17.9% and 18.0% CanL seroprevalences, respectively (Table 1; Fig. 1 panel 1B). Within the Centro NUTS2 region, CanL seroprevalence ranged from 19.2% in Coimbra to 4% in Viseu-Dão-Lafões (Table 1; Fig. 1 panel 1B), with an overall CanL seroprevalence of 13.4% Table 1; Fig. 1 panel 1A). In Alentejo, CanL seroprevalence was 10.3% (Table 1; Fig. 1 panel 1A). Notable intra-NUTS2 variation was detected, ranging from 23.1% in Alto Alentejo to 7.1% in Alentejo Litoral (Table 1; Fig. 1 panel 1B). The Norte region showed the lowest overall seroprevalence (5.2%) (Table 1; Fig. 1 panel 1A), with values ranging from 0% in Alto Minho to high 20.7% in Douro (Table 1; Fig. 1 panel 1B). No statistically significant differences were observed for overall CanL distribution between littoral and interior regions of mainland Portugal (Table 1). The distribution of CanL sick seropositivity occurred indistinguishably between the interior and littoral regions of mainland Portugal (χ2 = 0.978, df = 1, *p* = 0.323; Table 1).

Regarding FeL, overall seropositivity was 5.0% (24/478) and sick FeL seropositive cats represented 3.6% (17/478) of the studied feline population. Individual seropositivity to each antigen ranged from 8.6% (41/478) for SPLA and 10.5% for rK39 to 11.1% for CPX. The geographical distribution of FeL seropositivity was stratified by NUTS2 and corresponding NUTS3 regions or municipalities and is described in Table 1 and Fig. 1 (panels 1C and 1D). A total of 478 cats were sampled, of which 277 (57.9%) were from the littoral regions and 201 (42.1%) from interior regions (Table 1). Sampling encompassed the Norte (20.5%), Centro (20.3%), Oeste e Vale do Tejo (13.2%), Lisboa (18.6%), Península de Setúbal (19.7%) and Alentejo (7.7%) (Table 1).

A uniform distribution of FeL seroprevalence was noted across Centro, Oeste e Vale do Tejo, and Alentejo NUTS2 regions (ranging from 7.2% to 8.1%, Table 1, Fig. 1 panel 2C), contrasting with a heterogeneous pattern of FeL distribution observed between different NUTS3 subregions or neighbouring municipalities. The highest recorded FeL seroprevalence was registered for NUTS3 regions within the Centro, including Aveiro (18.2%), followed by Beiras and Serra da Estrela (15.8%) (Table 1; Fig. 1 panel 1D). The NUTS3 regions of Baixo Alentejo (15.8%), Tâmega e Sousa (15.4%), Lezíria do Tejo and Médio Tejo (12.5%) presented similar FeL seroprevalence (Table 1; Fig. 1 panel 1D).

Overall, no statistically significant differences were observed for FeL distribution between the littoral and the interior regions (χ2 = 1.515, df = 1; *p* = 0.218; Table 1).

**Fig. 2:**
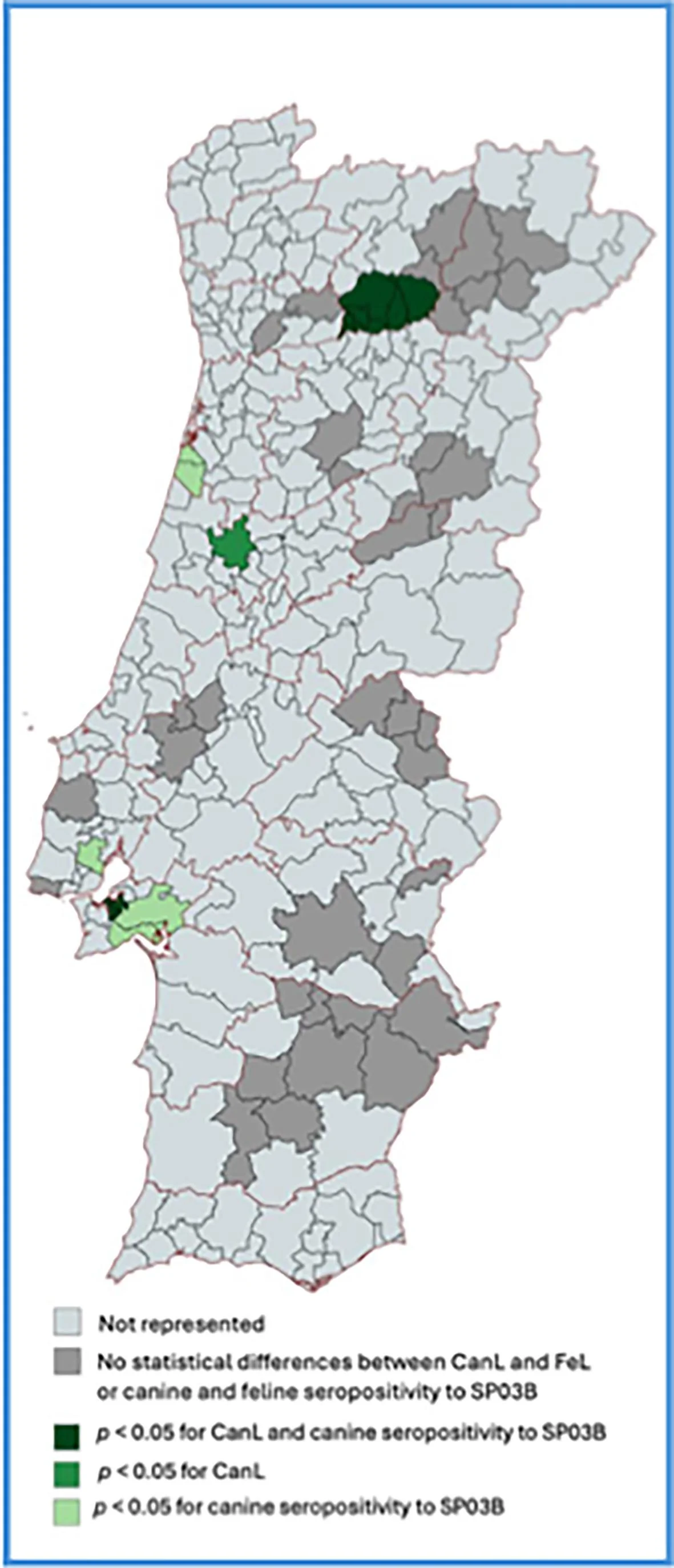
Assessment of exposure to *Leishmania infantum* and *Phlebotomus perniciosus* among sympatric dogs and cats from different regions of mainland Portugal.

A global analysis of CanL and FeL in mainland Portugal indicates a significantly higher prevalence of canine seropositivity compared to feline (*p* < 0.001) (Table 1; Fig. 1, panel 1). When examining sympatric animals sampled during the same visits, CanL seroprevalence surpassed that of FeL in the NUTS3 regions of Douro (*p* = 0.01) and Coimbra (*p* = 0.009), as well as in the municipalities of Barreiro and Moita (*p* = 0.032) (Table 1, Fig. 2).

### 3.2 Canine and feline susceptibility to Leishmania-associated disease

In total, 5.1% (*n* = 61/1194) of the canine population had a clinical and serological diagnosis of CanL. Besides, 6.7% (*n* = 80/1194) of the dogs tested seropositive but presented a regular physical exam (Table 1). Analysis of host factors (Table 2) revealed an even distribution of seropositivity between the different age groups (χ2 = 3.460, *df* = 5, *p* = 0.866), a statistically significant increase of seropositivity in male dogs compared to females (χ2 = 6.689, df = 1, *p* = 0.010) and those presenting suboptimal body condition scores (BCS, χ2 = 10.627, df = 1, *p* = 0.011). Among CanL sick dogs, seropositivity was significantly increase in the group of intact animals compared to the spayed or neutered ones (χ2 = 13.010, df = 1, *p* < 0.001).

**Table 2:** Association between signalment variables and canine (CanL) or feline (FeL) leishmaniosis as determined by an enzyme-linked immunosorbent assay against soluble promastigote *Leishmania* antigens (SPLA), *L. infantum* recombinant kinesin k39 (rK39) and L. *infantum* recombinant cytosolic peroxiredoxin protein (CPX). A two-proportion Chi-squared test was used to evaluate associations between signalment variables (age, sex, previous gonadectomy, having a specific breed and body condition score [BCS]) and serologic results in both canine and feline populations. Statistically significant differences were considered for *p* < 0.05.

Variable	No. of CanL^a^ seropositive dogs (%) per studied variable	No. of sick CanL^a^ seropositive dogs (%) per studied variable	No. of FeL^b^ seropositive cats (%) per studied variable	No. of sick FeL^b^ seropositive cats (%) per studied variable
Total seropositive (%)	141/1194 (11.8)	61/141 (43.3)	24/478 (5.0)	17/478 (3.6)
**Signalment variables**				
**Age**				
6 months – 1 year	5/52 (9.6)	2/52 (3.8)	1/19 (5.3)	1/457 (5.3)
1–3 years	25/273 (9.2)	7/273 (2.6)	7/252 (2.8)	3/252 (1.2)
4–8 years	87/689 (12.6)	38/689 (5.5)	14/159 (8.8)	11/159 (6.7)
9–12 years	19/135 (14.1)	11/135 (8.1)	1/19 (5.3)	1/19 (5.3)
>12 years	5/43 (11.6)	3/43 (7.0)	1/8 (12.5)	1/8 (12.5)
	χ2 = 3.460, *df* = 5 *p* = 0.866	χ2 = 7.529, *df* = 5 *p* = 0.184	χ2 = 9.486, *df* = 5, *p* = 0.148	χ2 = 12.314, *df* = 5, *p* = 0.031*
**Sex**				
Male	94/674 (13.9)	46/674 (6.8)	16/207 (7.7)	13/207 (6.3)
Female	46/509 (9.0)	15/509 (2.9)	8/248 (3.2)	4/248 (1.6)
	χ2 = 6.689, *df* = 1 *p* = 0.010*	χ2 = 9.069, *df* = 1 *p* = 0.003*	χ2 = 4.585, *df* = 1, *p* = 0.032*	χ2 = 6.911, *df* = 1, *p* = 0.009*
**Gonadectomy**				
Yes	81/706 (11.5)	23/706 (3.3)	21/280 (7.5)	15/280 (5.4)
No	57/459 (12.4)	37/459 (8.1)	3/192 (1.6)	2/192 (1.0)
	χ2 = 0.215, *df* = 1 *p* = 0.643	χ2 = 13.010, *df* = 1 *p* < 0.001*	χ2 = 8.175, *df* = 1 *p* = 0.004*	χ2 = 6.323, *df* = 1, *p* = 0.012*
**Breed** 1186/1194				
Mongrel	100/914 (10.9)	19/914 (7.0)	24/478 (5.0)	17/478 (3.6)
Pure- or mix breeds^c^	40/272 (14.7)	41/272 (4.5)	NO	NO
	χ2 = 2.912, *df* = 1 *p* = 0.088	χ2 = 2.177, *df* = 1 *p* = 0.140		
**BCS**^d^				
I/V or II/V	28/137 (20.4)	25/137 (18.2)	6/71 (8.5)	5/71 (7.0)
III/V to *V*/V	113/1044 (10.8)	36/1044 (3.5)	18/358 (5.0)	5/358 (1.4)
	χ2 = 10.627, *df* = 1, *p* = 0.011*	χ2 = 52.992, *df* = 1, *p* < 0.001*	χ2 = 1.375, *df* = 1, *p* = 0.241	χ2 = 8.157, *df* = 1, *p* = 0.004*

a CanL is represented by seropositive dogs to SPLA, rK39 or both.

b FeL is represented by SPLA, rK39 and CPX seropositive cats.

c Identified pure or cross breeds include: Cocker spaniel (*n* = 1), Dougue Bordeux (*n* = 1), English bulldog (*n* = 1), Epagnol Breton (*n* = 4), French Bulldog (*n* = 1), Galgo (*n* = 1), German-shepherd (*n* = 3), Irish Setter (*n* = 1), Labrador Retriever (*n* = 6), Pinscher (*n* = 1), Portuguese Podengo (*n* = 11), Portuguese Perdigueiro (*n* = 4), Pug (*n* = 1), Portuguese Rafeiro do Alentejo (*n* = 2), Shar-Pei (*n* = 1), Staffordshire Terrier (*n* = 3).

d BCS: five body condition scores were considered, from I to V. For convenience, BCS I and BCS II were grouped together representing famished, frail or underweight animals (cachectic [BCS I] and thin [BCS II]). BCS III to V were grouped together to represent animals without signs of sub nutrition, including those with an ideal body condition (BCS III), slightly overweight (BCS IV) and obese (BCS V).

Sick seropositive dogs represented 43.3% (61/141) of the total CanL. The clinical signs most frequently associated to seropositivity also showed statistically significant differences compared to their occurrence in the general CanL-seronegative population (*p <* 0.001). These included dermatological lesions (33.3%), lymphadenopathy (22.7%), and poor body condition or low BCS (19.9%). Ophthalmic lesions (9.9%), pale mucous membranes (9.9%) and lesions of the mucocutaneous transitions (5.7%) were the second most registered physical examination findings (Table 3).

**Table 3:** Associations between canine (CanL) and feline (FeL) leishmaniosis or seropositivity to *Phlebotomus perniciosus* salivary protein (SP) 03B and clinical signs of disease. Chi-square tests were used to evaluate associations between clinical variables and serologic results in both canine and feline populations. Fisher's Exact Test (FET) was applied when expected frequencies were below 5. Statistically significant differences were considered for *p* < 0.05.

Health variables	No. of CanL seropositive dogs^h^ (%)	No. of FeL seropositive cats^i^ (%)	No. of SP03B seropositive dogs (%) per studied variable	No. of SP03B seropositive cats (%) per studied variable
Total seropositive	141/1194 (11.8)	24/465 (5.2)	181/995 (18.2)	21/333 (6.3)
Visibly sick	61 (21.4) χ^2^ = 31.890, df = 1, *p* < 0.001*	17 (13.1) χ^2^ = 20.908, df = 1, *p* < 0.001*	51/181 (28.2) χ^2^ = 2.850, df = 1, *p =* 0.091	8/21 (38.1) χ^2^ = 0.132, df = 1, *p* = 0.716
**Clinical signs and lesions**				
Systemic signs of disease^a^	23 (16.3) χ^2^ = 21.166, df = 1, *p* < 0.001*	11 (45.8) *p*#*<* 0.001*	16/181 (8.8) χ^2^ = 1.858, df = 1, *p* = 0.173	4/61 (6.6) *p*# = 1.0
Lymphadenopathy	32 (22.7) χ^2^ = 44.920, df = 1, *p <* 0.001*	0	20/181 (11.0) χ^2^ = 7.360, df = 1, *p* = 0.007*	0
Pale mucous membranes	14 (9.9) χ^2^ = 16.431, df = 1, *p <* 0.001*	7 (41.2) *p*# = 0.004*	9/181 (5.0) χ^2^ = 0.591, *p* = 0.442	2/21 (9.5) *p*#*=* 1.0
Opthalmic lesions^b^	14 (9.9) χ^2^ = 12.158, df = 1, *p =* 0.001*	4 (16.7) *p*#*=* 0.013*	11/181 (6.1) χ^2^ = 2.080, df = 1, *p* = 0.149	2/18 (11.1) *p*#*=* 0.316
Skin lesions^c^	47 (33.3) χ^2^ = 59.829, df = 1, *p <* 0.001*	5 (20.8) *p*# = 0.041*	31/181 (17.1) χ^2^ = 5.274, df = 1, *p* = 0.020*	4/27 (14.8) *p*#*=* 0.079
Neurological signs	0 (0) *p*# = 0.06	1 (4.2) *p*#*=* 0.147	2/181 (1.1) *p*# = 1.0	1/3 (33.3) *p*#*=* 0.178
Gastro-intestinal signs	0 (0) *p*# = 1.0	3 (12.5) *p*#*=* 0.015*	0/181 (0) *p*# = 1.0	0/10 (0) *p*#*=* 1.0
Uro-genital signs^d^	2 (1.4) *p*# = 0.380	1 (4.2) *p*#*=* 0.101	2/181 (1.1) *p*# = 0.300	0/2 (0) *p*#*=* 1.0
Musculoskeletal lesions^e^	9 (6.4) χ^2^ = 1.075, df = 1, *p* = 0.234	NO	10/181 (5.5) χ^2^ = 0.135, df = 1, *p* = 0.713	NO
Respiratory^f^	0 (0) *p*# = 1.0	4 (16.7) *p*#*=* 0.010*	2/181 (1.1) *p*#*=* 0.642	0/16 (0) *p*#*=* 0.611
Lesions of the mucocutaneous transitionsg1^,^g2	8 (5.7) *p*# = 0.052	7 (29.2) *p*#*=* 0.005*	5/181 (2.8) *p*#*=* 0.794	3/39 (7.7) *p*#*=* 0.723
Underweight	28 (19.9) χ^2^ = 10.148, df = 1, *p* = 0.001*	6 (25.0) *p*# = 0.264	31/181 (17.1) *p =* 0.021*	4/60 (6.7) *p*#*=* 1.0

ELISA, enzyme-linked immunosorbent assay; CPX*, L. infantum* recombinant cytosolic peroxiredoxin protein; rk39, *L. infantum* recombinant kinesin k39; SPLA, soluble promastigote *Leishmania* antigens; NO, not observed; SP03B, recombinant salivary protein 03B from *P. perniciosus; df* = 1 for all Chi-square (χ^2^) measurements.

a Systemic signs of disease include at least one of the following: fever, inappetence, dehydration, underweight/ poor body condition, anaemia/pale mucous membranes.

b Ophthalmic lesions, include at least one of the following: conjunctivitis, keratitis, corneal lesions, periocular alopecia, blepharitis.

c Skin lesions include at least one of the following: alopecia, crusts, ulcers, wounds, lesions of the pinna, hyperkeratosis, furfuraceous dermatitis.

d Urogenital signs include at least one of the following: vulvar discharge, pyometra; ulceration of the penile mucosa/ balanoposthitis.

e Muscular-skeletal lesions include at least one of the following: generalized muscle atrophy; atrophy of the temporal and zygomatic muscles.

f Respiratory signs include at least one of the following: reverse sneezing, sneezing.

g1 Canine lesions of the mouth, including periodontal disease; crusts and/or ulceration of the mucocutaneous transitions; rhinitis and ulceration of the nostrils; crusts and/or ulceration and/or hyperkeratosis of the muzzle; depigmentation of the muzzle; periodontal disease.

g2 Feline lesions of the mouth, including ulcers, gingivitis, glossitis, mucositis, and lesions of the muzzle, including ulcer and hyperkeratosis.

# FET, Fisher exact test.

h CanL is represented by dogs seropositive to SPLA and/or rK39.

i FeL is represented by cats seropositive to SPLA, rK39 and CPX.

Analysis of feline host factors (Table 2) excluded positive associations between FeL seropositivity and age group (χ2 = 9.486, df = 5, *p* = 0.148; Table 2). If only FeL seropositive sick cats are compared, then statistically significant differences are recorded (χ2 = 12.314, df = 5, *p* = 0.031) and cats over 12 years of age are more frequently ill. FeL seropositive male cats were significantly over-represented compared to females (χ2 = 4.585, df = 1, *p* = 0.032). In FeL sick cats, a suboptimal BCS was statistically associated to seropositivity (χ2 = 8.157, df = 1, *p* = 0.004). Gonadectomy was found statistically associated to FeL seropositivity in both FeL sick (χ2 = 6.323, df = 1, *p* = 0.012) and apparently healthy cats (χ2 = 8.175, df = 1, *p* = 0.004).

FeL seropositivity was statistically associated with systemic and unspecific signs of disease (*p <* 0.001; e.g. mucous membranes pallor, poor body condition); ophthalmic (*p =* 0.013) and dermatological lesions (*p* = 0.041); gastro-intestinal signs (*p =* 0.015); respiratory signs (*p =* 0.010), and oral lesions (Table 3).

### 3.3 Serological assessment of exposure to Phlebotomus perniciosus bites in cats and dogs

Exposure to *P. perniciosus* was assessed in dogs (*n* = 995) and cats (*n* = 335) and its distribution was evaluated across mainland Portugal (Table 1, Fig. 1 panel 2).

**Fig. 3:**
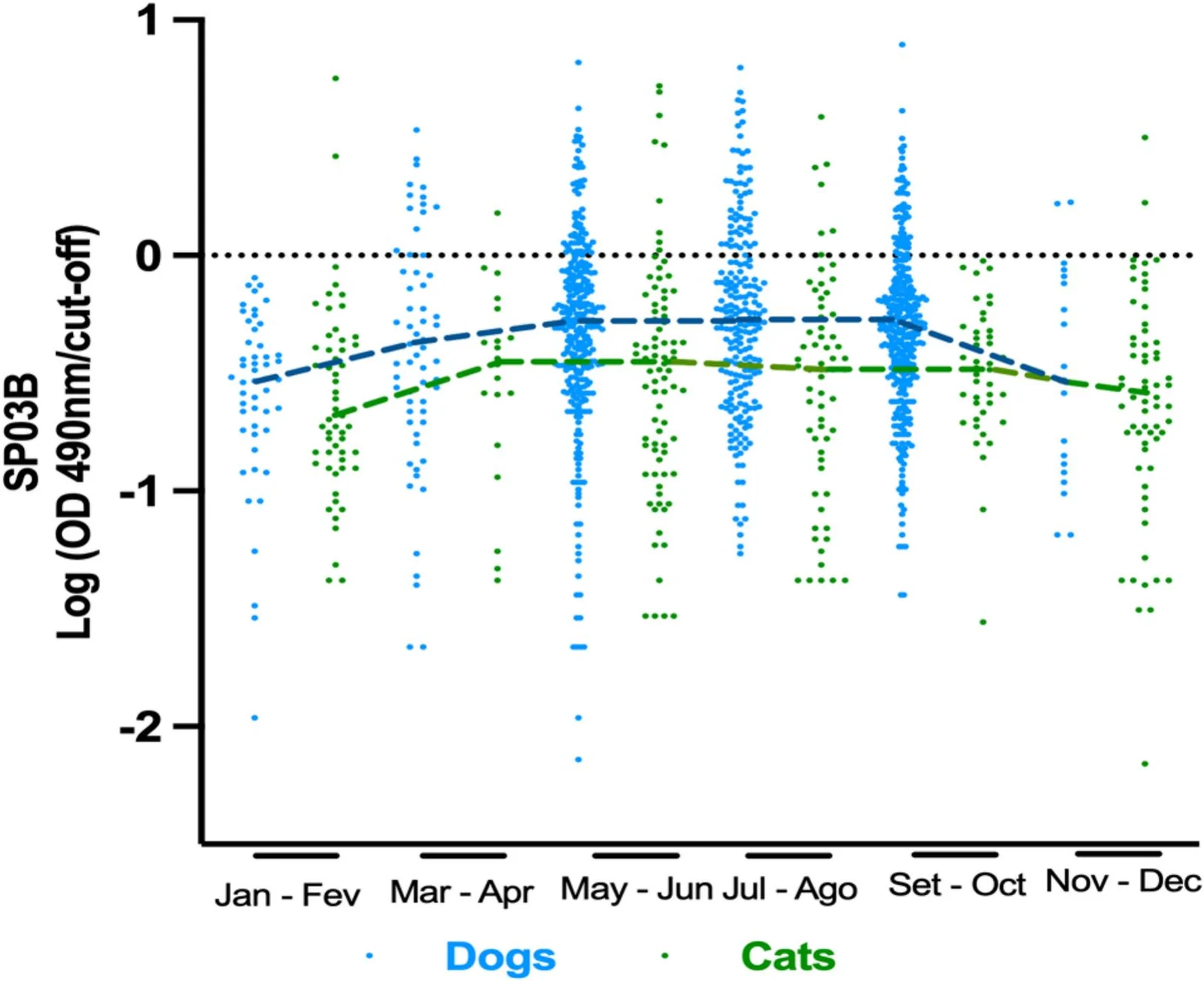
Distribution of canine and feline exposure to *Phlebotomus perniciosus* salivary protein (SP) 03B according to month of sampling. Exposure was assessed through antibody detection against SP03B using an enzyme-linked immunosorbent assay (ELISA). Monthly distribution of SP03B reactivity in dogs (*n* = 995) and cats (*n* = 335). Each dot represents the average optical density (OD) measured at 490 nm and normalized to the cut-off for each individual animal. The Y axis displays OD values on a log10 scale. The horizontal black dotted line represents the cut-off value for SP03B antigen. The blue and green dashed line represents the median OD values for dogs and cats, respectively. (For interpretation of the references to colour in this figure legend, the reader is referred to the web version of this article.)

In dogs, median OD values for IgG to SP30B were highest between May and October, with a progressive increase beginning in March/April and a marked decline from September/October. No canine-SP03B seropositive samples were detected between January and February (Fig. 3). The Centre and Lisbon NUTS2 regions presented the highest canine exposure to *P. perniciosus* (21.4% and 24.8%, respectively), with seropositivity to SP03B ranging from 13.0% in the North to 24.8% in Lisbon (Table 1; Fig. 1 panel 2A). A marked heterogeneity was evident at the NUTS3 level (Table 1; Fig. 1 panel 2B), with seropositivity to SP03B surpassing 40% among dogs from Douro, Aveiro and Lezíria do Tejo.

In cats, SP03B median seroreactivity remained relatively constant from March/April to September/October, with lower median values from November to February (Fig. 3). Feline seropositivity to SP03B varied from 0% in Alentejo to 9.4% in the Oeste e Vale do Tejo regions (Table 1). The Norte, Centro and Oeste e Vale do Tejo NUTS2 regions showed similar frequencies of exposure to *P. perniciosus* (8.4%, 8.2% and 9.4%, respectively) (Table 1). Marked heterogeneity was found at the level of NUTS3 or municipalities, with exposure ranging from 0% in all three subregions of Alentejo to 37.5% in Lezíria do Tejo (Table 1, Fig. 1 panel 2).

Statistically significant differences were registered for SP03B seropositivity between the studied canine and feline populations (20.2% versus 6.2%, *p* < 0.001) and between sympatric animal species living in the NUTS3 regions of Douro (*p* = 0.001) and Aveiro (*p* = 0.003), or the municipalities of Loures (*p* = 0.005), Barreiro and Moita (*p* = 0.04), Palmela or Setúbal (*p* = 0.025) (Table 1; Fig. 1 panel 2B and 2C).

## 4 Discussion

In Portugal, zoonotic leishmaniosis by *L. infantum* is endemic and the country relies on passive case detection in both humans and dogs to sustain a surveillance strategy. To address the need to improve surveillance among *L. infantum* main reservoirs for human infection, a field- and laboratory-integrated epidemiological approach was implemented, proposing the national CROA network as a surveillance platforms, and exploring the sentinel potential of unowned dogs and cats. This strategy allows a scalable sampling of a highly exposed population of animal reservoirs, such as free roaming dogs and cats. It combines a multi-antigen ELISA to monitor *Leishmania* infection and SP03B-based serology as a marker of vector-host contact. The data collected from two different host species within the geographical resolution of NUTS2/NUTS3/municipal regions enhances granularity of epidemiological analysis and exposes different ecological interactions between each host species, *Leishmania* parasites and *P. perniciosus* vectors.

In leishmaniosis surveillance, the lack of a universally accepted serological gold standard and the existence of various and different commercially available or in-house produced tests leads to epidemiological reports that reflect diagnostic selections, driven by commercial availability and/or laboratory convenience. This complicates standardisation and comparison. Nevertheless, serology remains a cornerstone of surveillance because the disease is strongly associated with specific antibody responses [25], [26], [29]. The introduction of recombinant antigens, notably kinesin-derived rK39, has improved diagnostic accuracy over earlier methods such as DAT or IFAT [30]. Additionally, combining multiple antigens in a single ELISA improves diagnostic value for both CanL [24], [25] and FeL [26]. The antigen-specific ROC analyses and data-driven cut-offs supported a diagnostic rule of SPLA and/or rK39 for CanL. This multi-antigen strategy achieved higher diagnostic performance with CanL diagnostic sensitivity and specificity reaching 99% and 98%, respectively. The FeL classification followed established multi-antigen criteria [26]. Although internal validation confirmed the high diagnostic accuracy of the multi-antigen ELISA, independent external validation remains an essential next step before widespread operational rollout. This requirement would support the choice of diagnostic methodology herein implemented, inter-laboratory reproducibility, operational performance, and applicability under high throughput screening conditions.

ELISAs using vector salivary antigens information on recent exposure to sandfly bites and may be useful for assessing geographical patterns of vector–host contact [[12], [31], [32], [33], [34]]. Antigens such as SP03B elicit strong antibody responses and allow laboratory-based surveillance without the need for salivary gland dissections to obtain crude antigens [27], [33], [34]. The choice of salivary antigens should be tailored to local phlebotomine ecology and does not replace xenomonitoring (phlebotomine species identification, abundance, and infection rates). In areas where multiple competent vectors co-circulate, antigens from different *Phlebotomus* spp. should be ideally included. However, the availability of recombinant proteins can limit this approach [32]. Nevertheless, given the high degree of conservation for the yellow related proteins of the different species included in the Larroussius subgenus, cross reactivity between *P. perniciosus* and *P. ariasi* is expected in this study.

CanL seroprevalence showed marked geographical heterogeneity, with highest values around the Tagus River Valley, including Lezíra do Tejo and Médio Tejo, the Douro region in the Norte, Coimbra region in the Centro, and the municipalities composing Lisboa NUTS2 region. Compared to prior regional or national CanL serosurveys conducted in Portugal (references [[12], [13], [35]] as briefly summarized in Supplementary Fig. 2) our findings are consistent with established trends regarding the absence of CanL seropositivity and the identification of subregions with increased canine exposure or infection by *Leishmania* (subregion of Alto Alentejo, subregion of Beiras e Serra da Estrela, subregion of Douro, subregion of Coimbra and Lezíria do Tejo as examples). Furthermore, there is trend for higher exposure to *Leishmania* in regions where seropositivity to SP03B is increased. Despite no statistically significant differences were found between CanL or FeL seroprevalence between the coastal urban regions and the more rural settings in the interior of the country. However, data on human VL cases diagnosed between 2010 and 2020 in public Portuguese hospitals point out to a rural shift of distribution of the VL incidence [5].

FeL seropositivity was lower than CanL seropositivity at NUTS2 regional stratification, yet more heterogeneous at NUTS3/municipal scales, revealing micro-foci that a broader analysis might miss. These patterns justify the need for surveillance and application of targeted preventive and control measures at sub-national levels.

SP03B responses revealed a clear seasonal pattern in dogs, rising from March/April to a peak between May/April and September/October, then declining. No SP03B seropositive samples were identified between January and February. This pattern aligns with known sandfly activity in Portugal (May–November) [[12], [36]]. Dogs were more frequently SP03B-seropositive than cats in geographical and time-matched comparisons. Such differences were statistically significant in most NUTS2 regions, suggesting ecological differences in host- vector dynamics. Regional variations may also reflect transient vector community composition and density. For example, *P. sergenti* has been reported to be more prevalent than *P. perniciosus* in certain areas of Alentejo [37], which could explain the 0% feline SP03B reactivity detected there, despite the 19.8% reactivity found in dogs.

Most seropositive dogs were apparently healthy, highlighting a substantial subclinical reservoir that would be missed by clinical case-based surveillance alone. Clinical correlates – poor body condition, lymphadenopathy, mucous membrane pallor, ophthalmic and dermatological lesions, and underweight – were associated with CanL seropositivity and support WOAH case definitions [29] reinforcing their value for triage and differential diagnosis in CROA workflows.

It is still unclear if cats can sustain the *L*. *infantum* transmission cycle in the absence of dogs, particularly given lower FeL prevalence within the same endemic region. Despite lower sentinel sensitivity in cats, feline surveillance has added granularity to epidemiological data on certain micro-foci and should be used as a complementary component of a CROA – TNR strategic collaboration.

The geographical distribution of CanL seropositivity observed herein shares overall trends with data previously reported for owned dogs, supporting the potential value of the CROA network as a complementary sentinel population for leishmaniosis surveillance. In a new paradigm for the control of leishmaniosis, and other vector-borne diseases, surveillance should not be restricted to a single group of dogs sharing similar lifestyles but should instead leverage different dog populations as complementary sentinels. Furthermore, the recruitment of veterinarians to support canine and feline sampling for epidemiological studies has largely depend on interest and good will of those contributing to such research studies [13]. Instead, the proposed approach should rely on the MV workforce, largely guided by recommendations from the national veterinary authorities and legislation.

While stray dog populations are often an underestimated problem with virtually non-existent governmental management protocols [17], Portugal CROA network has long been part of a national program for the control of zoonotic diseases, but not for leishmaniosis. The CROAs receive dogs and cats from numerous municipalities within a region, including animals that traverse urban, peri-urban, and rural zones. Consequently, these animals provide valuable targets to assess exposure, infection, and potential transmission of diverse pathogens over a broad geographical area – beyond private properties or the usual ranges of owned animals. Within participating CROA facilities, animals were randomly selected among those eligible for inclusion. Despite bias associated to sampling of unowned dogs, including the impossibility to draw a full clinical history or past traveling/relocation experiences, this still enabled the selection of animals with a great variety of sociodemographic backgrounds, bringing this population closer to the real-world epidemiological scenario of vector-borne diseases transmission.

The CROA-based approach provides a scalable framework for geographically distributed sampling of animal reservoirs across NUTS2/NUTS3/municipal levels. Although, the present cross-sectional study does not allow assessment of temporal trends or seasonal changes in infection or vector exposure, the repeated implementation of standardized sampling could, in the future, provide longitudinal information on *L. infantum* circulation and vector exposure. The data generated revealed infections/exposure hotspots. This capacity is a clear demonstration that this approach can contribute to the prioritization of hotspots for integrated interventions. These can be easily implemented into national strategies to tackle leishmaniosis, including promotion of health education, responsible pet ownership and awareness campaigns to incentivise the implementation of preventive measures against sandfly exposure. It also enables the prioritization of hotspots for integrated interventions that can be easily implemented into national strategies to tackle leishmaniosis, including promotion of health education, responsible pet ownership and awareness campaigns to incentivise the implementation of preventive measures against sandfly exposure.

The lack of a clear littoral-interior gradient suggests that micro-ecological and management factors are more important than the major geographical and climatic differences, confirming the need for detailed risk assessments.

The main limitations of this approach are the typical of operational surveillance. Although animals were randomly selected among eligible individuals within participating CROA facilities, the participating facilities themselves were recruited on a voluntary basis. Therefore, facility-level selection bias cannot be excluded, and national representativeness cannot be assumed. Populations in shelters may differ from the owned-dog population, selection bias due to convenience in sampling at different sites cannot be fully controlled, and we face unequal sample size across geographical regions. These specific constraints can be overcome if sampling is systematically performed at the animals' admission. Expanded molecular testing by PCR and entomological corroboration should be considered as complementary approaches. PCR may provide valuable complementary information, particularly in seronegative animals or in cases of subclinical infection. However, the diagnostic sensitivity of PCR performed on blood samples is limited, whereas the preferred matrices (bone marrow or lymph node aspirates) require invasive sampling procedures that are less suitable for large-scale epidemiological surveys. Moreover, combining serological and molecular testing in a nationwide surveillance program would substantially increase logistical complexity (namely invasiveness associated to the collection of bone marrow and/or lymph node aspirates from dogs and cats; training and equipment required to perform DNA extraction and amplification) and costs, potentially compromising its operational feasibility. These practical considerations supported the use of a validated serological approach as the primary diagnostic tool in the present study.

Although the present study focuses on veterinary surveillance, its implementation is intended to contribute to a broader One Health surveillance framework. Performing this field sampling, combined with centralized laboratory diagnosis, could contribute to future assessment of spatial patterns of infection. Furthermore, if collected prospectively through repeated sampling, might also support the evaluation of temporal changes. Ultimately, this approach can generate systematic, harmonized data on *L. infantum* infection and exposure in domestic reservoirs, that can be routinely shared with the competent veterinary and public health authorities. Such information would support integrated risk assessment and complement the surveillance data obtained from the already established program for VL notification, vector surveillance (e.g. Rede de Vigilância de Vetores, REVIVE), and environmental monitoring as these become available. While epidemiological thresholds for triggering specific interventions remain to be established within national surveillance guidelines, the proposed approach provides a structured foundation that could be integrated into emerging interoperable surveillance platforms, such as the project for an Integrated Surveillance and Early Warning System for Zoonoses (SIVIZ), thereby strengthening future One Health surveillance of leishmaniosis.

## 5 Conclusions

This study demonstrates the feasibility of using CROA facilities as a geographically distributed framework for collecting epidemiological and serological data on zoonotic leishmaniosis in Portugal. This approach highlights the potential of these public infrastructures to provide geographically distributed epidemiological information that may contribute to future risk assessment and surveillance activities to support One Health approaches to leishmaniosis control. The proposed framework represents a scalable model that could be further developed and validated in Portugal and other endemic Southern European settings.

## Use of artificial intelligence tools

No generative AI or AI assisted Technologies were employed in data analysis or during the manuscript preparation.

## Other notes/disclaimers

Nothing to declare.

## CRediT authorship contribution statement

**Clara M. Lima:** Writing – original draft, Methodology, Investigation, Formal analysis, Data curation, Conceptualization. **Luís Cardoso:** Writing – review & editing, Supervision, Formal analysis, Conceptualization. **Joana Tavares:** Writing – review & editing, Funding acquisition, Formal analysis. **Mariana Portugal:** Writing – review & editing, Resources, Investigation. **Rita Gordo:** Writing – review & editing, Resources, Investigation. **Rui Ferreira:** Writing – review & editing, Resources, Investigation. **Nuno Santarém:** Writing – review & editing, Validation, Methodology, Investigation, Formal analysis, Conceptualization. **Anabela Cordeiro da Silva:** Writing – review & editing, Supervision, Funding acquisition, Conceptualization.

## Ethics declaration

This study was conducted in accordance with the following guidelines for animal welfare and/or reporting: Not Applicable.

This study was approved by the Orgão Responsável pelo Bem Estar Animal (ORBEA) do Instituto de Ciências Biomédicas Abel Salazar - Universidade do Porto, Porto, Portugal.

(Approval No. Project number: 385/2020/ORBEA)

## Funding sources

This work was funded by Fundação para a Ciência e Tecnologia (FCT)/ Ministério da Educação e Ciência (MEC) and FEDER through the research Unit No. 4293 and the PhD scholarship 2020. 07306.BD. Nuno Santarém received funding as an assistant researcher by national funds through FCT and co-funded through the European Social Fund within the Human Potential Operating Program with reference 2021.04285.CEECIND/CP1663/CT0004.

The work also received financial support through ELANCO Animal Health Portugal. The role of the sponsor was limited to partially funding the project. The views expressed in this paper are those of the authors only, and do not necessarily represent or reflect the positions of ELANCO. Funders had no involvement in the study design, data collection, data analysis, data interpretation and discussion or conclusions.

## Declaration of competing interest

Rita Gordo is employed by Elanco Animal Health, Portugal, that partially financed the study but declares to have no financial or non-financial interests directly or indirectly related to the work presented in this study. She participated in field work, supporting with animal sampling and clinical evaluation, besides reviewing the manuscript. All other authors declare no conflict of interest related to this study.

## Data Availability

CL, NS, and ACS had full access to all the data and take responsibility for the integrity and accuracy of the data analysis. Raw data can be made available upon request.
