# Supplementary material for: Strategic surveillance plan for zoonotic leishmaniosis in Portugal
Source: One Health. 2026 Sep 10;23:101572. doi: 10.1016/j.onehlt.2026.101572 (PMC13602739; doi:10.1016/j.onehlt.2026.101572)
Supplement: Supplementary file 1 [file mmc1.docx]

**Supplementary Figure S1.** Demographic and clinical characteristics of canine leishmaniosis (CanL) positive (Group A) and negative (Group B) control groups.

Group A (n = 81) consisted of dogs living in various regions of Portugal where CanL is endemic. These dogs were admitted to a veterinary consultation under the following scenarios (i) presentation of clinical signs compatible with the disease, (ii) positive CanL screening prior to vaccination against CanL (iii) irregular health examination, haematological and/or serum biochemistry analysis and a positive parasitological test for *Leishmania* infection. Furthermore, CanL was diagnosed following laboratory assessment by means of at least one positive commercially available test, including serologic and/or direct parasitological tests, validated for CanL diagnosis.

Group B (n = 125) consisted of apparently healthy non-*Leishmania*-infected dogs from distinct geographical regions: Azores Islands (AZO, n = 38), a non-endemic archipelago in Northen Atlantic ocean hemisphere; Republic of Ireland (IRL), a non-endemic country in Europe (n = 30); Catalonia region in Spain (ESP, n = 28), an endemic region in Mediterranean coast, and a region of Porto metropolitan area in Portugal, close to the Atlantic coast (PT, n = 29).

The group of dogs from AZO included companion animals from Ilha Terceira (n = 5) and farm and cattle dogs from the Island of São Miguel (n = 33), all sampled during veterinary visits. None of the animals had a history of traveling outside the Archipelago. Left over volume of samples from clinical laboratory work were used in this study and following the owner´s consent. A blood-PCR using RV1 and RV2 primers targeting *Leishmania* kinetoplastid DNA (following the protocol described by Lima CM et al., 2024) was negative for all AZO samples.

Dogs from IRL, ESP and PT participated in veterinary blood donation programmes, following dedicated guidelines and protocols. All blood donor dogs had a negative real-time PCR (RT-PCR; EXOone Leishmania, Exopol, Zaragoza, Spain) performed with whole blood-genomic DNA (automated extraction by Roche MagNAPure LC 2.0 System DNA Extractor using Roche extraction kits MagNAPure LC DNA Isolation Kit I, Roche Diagnostics International AG, Rotkreuz, Switzerland) and a negative serological result by two independent tests validated for CanL diagnosis (SNAP *Leishmania*, Idexx, UK; VetLine *Leishmania* in-house ELISA, Gold Standard Diagnostics, Frankfurt, Germany). The *Leishmania*-free status of the group of dogs from ESP and PT was followed up for a minimum of four blood donations (approximately 2 years) beyond this study. Animals were later withdrawn from blood donation programs for reasons unrelated to health status or infectious disease.
