# Supplementary material for: Strategic surveillance plan for zoonotic leishmaniosis in Portugal
Source: One Health. 2026 Sep 10;23:101572. doi: 10.1016/j.onehlt.2026.101572 (PMC13602739; doi:10.1016/j.onehlt.2026.101572)
Supplement: Supplementary file 2 [file mmc2.docx]

**Supplementary Figure S2.** Figure 2. Geographical distribution of canine leishmaniosis (CanL) seroprevalence (%) in the studied population of dogs (n = 1194). Seroprevalence data was produced using an enzyme-linked immunosorbent assay and is depicted by geographical regions of mainland Portugal, classified according to the Nomenclature of Territory Units for Statistics (NUTS) classification 3, and the corresponding surveyed municipalities. Seropositivity was determined by an enzyme linked immunosorbent assay (ELISA). CanL was considered if samples tested positive to soluble promastigote *Leishmania* antigens (SPLA) and/or *Leishmania infantum* recombinant kinesin (rK39). Data on CanL regional distribution was compared to the findings from other studies reporting CanL distribution in mainland Portugal.
