# Supplementary material for: Strategic surveillance plan for zoonotic leishmaniosis in Portugal
Source: One Health. 2026 Sep 10;23:101572. doi: 10.1016/j.onehlt.2026.101572 (PMC13602739; doi:10.1016/j.onehlt.2026.101572)
Supplement: Supplementary file 3 [file mmc3.docx]

**Supplementary Table** **S1.** Descriptive statistics of seroreactivity against soluble promastigote *Leishmania infantum* antigens (SPLA) and *L. infantum* recombinant kinesin k39 (rk39). Optical density (OD) values were determined by enzyme-linked immunosorbent assay (ELISA) for group A (positive controls, n = 81) and group B (negative controls, n = 125). Differences between the mean rank OD values of Group A and Group B were determined by Mann-Whitney test, considering significant differences whenever *p* < 0.005.

| ELISA antigens | SPLA (OD) | |  | rK39 (OD) | |
| --- | --- | --- | --- | --- | --- |
| Control groups | A | B |  | A | B |
| Minimum | 0.018 | 0.003 |  | 0.022 | 0.003 |
| Maximum | 1.320 | 0.120 |  | 3.130 | 0.128 |
| Range | 1.300 | 0.117 |  | 3.110 | 0.125 |
| 25^th^ percentile | 0.159 | 0.019 |  | 0.409 | 0.016 |
| Median | 0.324 | 0.031 |  | 1.210 | 0.026 |
| 75^th^ percentile | 0.616 | 0.043 |  | 1.840 | 0.037 |
| Mean | 0.425 | 0.033 |  | 1.190 | 0.029 |
| Standard deviation | 0.317 | 0.019 |  | 0.854 | 0.019 |
|  | *p* < 0.001 | |  | *p* < 0.001 | |
