# Supplementary material for: Strategic surveillance plan for zoonotic leishmaniosis in Portugal
Source: One Health. 2026 Sep 10;23:101572. doi: 10.1016/j.onehlt.2026.101572 (PMC13602739; doi:10.1016/j.onehlt.2026.101572)
Supplement: Supplementary file 4 [file mmc4.docx]

**Supplementary Table** **S2.** Receiver Operating Characteristic (ROC) curve analysis determined for soluble promastigote *Leishmania* antigen (SPLA) and *L. infantum* recombinant kinesin k39 antigen (rk39), considering the canine leishmaniosis (CanL) negative control group B (n = 125) and the CanL positive control group A (n = 81).

| **Test** | **SPLA** | **rK39** | **SPLA and/or rK39** |
| --- | --- | --- | --- |
| **Cut-off** | 0.080 | 0.083 |  |
| **AUC** | 0.982 | 0.983 |  |
| **Sn (%)** | 95 | 94 | 99 |
| **Sp (%)** | 98 | 99 | 98 |
| **Sn+Sp (%)** | 193 | 193 | 196 |

AUC, area under the curve; Sn, sensitivity; Sp, specificity; Ag(s), antigen(s)
